# Proper housing conditions in experimental stroke studies—special emphasis on environmental enrichment

**DOI:** 10.3389/fnins.2015.00106

**Published:** 2015-03-30

**Authors:** Satu Mering, Jukka Jolkkonen

**Affiliations:** ^1^Lab Animal Centre, University of Eastern FinlandKuopio, Finland; ^2^Institute of Clinical Medicine - Neurology, University of Eastern FinlandKuopio, Finland

**Keywords:** cerebral ischemia, environmental enrichment, plasticity, rodent, sensorimotor functions

## Abstract

Environmental enrichment provides laboratory animals with novelty and extra space, allowing different forms of multisensory stimulation ranging from social grouping to enhanced motor activity. At the extreme end of the spectrum, one can have a super-enriched environment. Environmental enrichment is believed to result in improved cognitive and sensorimotor functions both in naïve rodents and in animals with brain lesions such as those occurring after a stroke. Robust behavioral effects in animals which have suffered a stroke are probably related not only to neuronal plasticity in the perilesional cortex but also in remote brain areas. There is emerging evidence to suggest that testing restorative therapies in an enriched environment can maximize treatment effects, e.g., the perilesional milieu seems to be more receptive to concomitant pharmacotherapy and/or cell therapy. This review provides an updated overview on the effect of an enriched environment in stroke animals from the practical points to be considered when planning experiments to the mechanisms explaining why combined therapies can contribute to behavioral improvement in a synergistic manner.

## Introduction

“No man is an island” (John Donne 1572–1631). Not only man, but also the behavior, phenotype, and responses of laboratory animals vary depending on the genotype of the animals and the housing conditions in which they are reared. The term “housing conditions” includes the physiological, chemical, and social environment as well as handling procedures and environmental enrichment. Scientists strive to standardize these conditions so that it is only the factor of interest which is changed in order to obtain both reliable and repeatable results from the minimum number of animals.

Wild rodents live in a very complex environment and they exploit a wide array of motor skills to explore their environment, find food, escape threats, in general to survive. In comparison, laboratory animals are considered to be housed in a very impoverished environment, quite often socially isolated. This raises the question about what are the proper housing conditions in which to study behavior in naïve animals or in animals after various brain insults. Another issue is whether the housing condition can affects or in the worst case scenario, even negate treatment effects?

The present review provides a short overview on housing conditions in laboratory rodent research with particular emphasis being placed on the possible contribution of an enriched environment to the spontaneous sensorimotor recovery after cerebral ischemia as well as that induced by restorative therapies.

## Law, regulations, and recommendations

The Guide for the Care and Use of Laboratory Animals (2011) states that “The primary aim of environmental enrichment is to enhance animal well-being by providing animals with sensory and motor stimulation, through structures and resources that facilitate the expression of species-typical behaviors and promote psychological well-being through physical exercise, manipulative activities, and cognitive challenges according to species-specific characteristics.”

According to the European legislation (Directive 2010/63/EU, ANNEX III), European Commission recommendations (2007/526/EC) and Finnish national legislation (Government Decree on the Protection of Animals Used for Scientific or Educational Purposes 564/2013), all animals should be provided with sufficient space of adequate complexity and they should be given a degree of control and choice over their environment. This may be achieved by using enrichment techniques, which are appropriate to the species-specific and individual needs. Furthermore, the enrichment practices must be regularly reviewed and updated.

In addition, several attempts have been conducted by stroke researchers to improve the quality of preclinical studies with the aim to prevent translational failures all too often encountered with neuroprotective compounds (Savitz et al., 2011; Dirnagl et al., 2013; Boltze et al., 2014). However, housing conditions have not yet been considered in any detail. The STEPS II guideline shortly states that “Consideration may also be given to applying clinically relevant rehabilitation to all treatment groups in functional testing” (Savitz et al., 2011).

## Housing conditions of laboratory animals—current practices

Rats have been used as laboratory animals since the end of 1800's. The most commonly used rat stocks like Wistar, Sprague-Dawley, and Long-Evans hooded rats were developed for research use in the beginning of the twentieth century (Koolhaas, 1999). Rats are generally considered as social animals, but they may also live a solitary existence (Weihe, 1987). They are highly adaptable but neophobic animals and breeding in laboratory conditions has led to the creation of tame animals which habituate easily and can be trained to tolerate even unpleasant procedures (Weihe, 1987). Even though it is recognized that laboratory rats differ from their wild conspecifics with respect to their behavioral and environmental demands (Inglis and Hudson, 1999), they still possess many of their natural needs such as the requirement for a safe, concealed area when resting (Hurst et al., 1999).

### The aim of environmental enrichment

The word “enrichment” carries a positive connotation, which can be seen in definitions like “modification of the environment resulting in an improvement in the biological functioning of captive animal” (Newberry, 1995) and “alteration to the living environment of captive animals in order to provide opportunities to express more of their natural behavioral repertoire” (Chamove, 1989; Van de Weerd and Baumans, 1995). In general, the goal is to increase the number and range of normal behaviors, to prevent or reduce development of abnormal behaviors, to increase the positive utilization of the environment and to improve the animal's ability to cope with behavioral and physiological challenges (Young, 2003). However, supplements which humans consider enrichment do not always confer beneficial effects on animals (Kaliste et al., 2006). The housing environment, including enrichment, may also increase (or decrease or have no effect at all) on the variation between animals and therefore impact on the number of animals needed in an experiment (Mering, 2000). This debate about the pros (e.g., better welfare and data quality) and cons (e.g., increased variability in test results) of standardization in relation to enrichment has been going on for years.

### Social enrichment

Environmental modifications or enrichment can be applied in several ways. For social animals, contact with other individuals ensures their welfare and acts as enrichment. Group or pair housing is recommended for social animals whenever possible (Directive 2010/63/EU, Commission recommendations 2007/526/EC). Group housing provides safety, competitors and companions offering complexity, changes in social relationships lead to unpredictability and appropriate breeding can make it possible to achieve these goals. These factors can be considered as the most important requirements of an animal of its environment, in order to satisfy its behavioral needs (Poole, 1992). When new groups are formed or reformed after some period of individual housing or isolation, animals must be carefully monitored, since aggression or other forms of adverse behavior may occur. If group housing is not possible, the importance of enrichment increases. Humans can also act as social enrichment for animals living in laboratory conditions.

### Spatial enrichment

**Figure 1:**
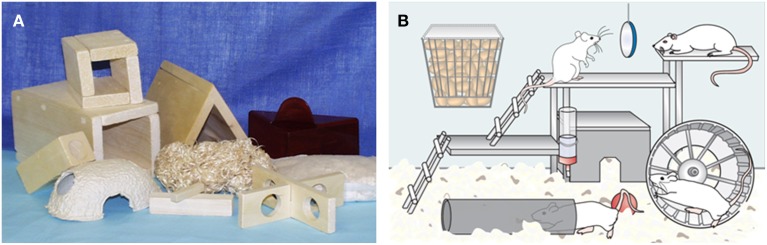
**Environmental enrichment. (A)** Different kinds of rodent enrichment (picture from Satu Mering). **(B)** Super-enriched environment provides multiple and complex stimuli to the animals (modified from Sivenius et al., 2002). Enriched environment usually consists of large cage to provide spatial stimuli, ladders and running wheel for sensorimotor and motor exercise and social interactions of 6–8 animals. In addition, replaceable objects such as toys provide novelty, all of which can enhance brain plasticity leading to improved cognitive and sensorimotor recovery after brain insults.

Nowadays a huge variety of commercial accessories are available (Figure 1A) and the housing environment can be spatially modified by using space and structural constructions. Adequate space is still a necessity for normal species-specific behavior and there are also regulations and recommendations that impose minimum floor areas for laboratory animals. The recommendations for the minimum floor areas are dependent on the weight of the animals, whether they are housed in groups, paired or singly, whether they are in stock, maintenance or undergoing experimental procedures or whether they are used for breeding (Guide for the Care and Use of Laboratory Animals, 2011; Directive 2010/63/EU ANNEX II; European Commission recommendations 2007/527/EC). Given this background, one can understand that implementation of enriched environment is challenging or impossible in the case of individually ventilated cages. Enrichment should not restrict the available space but rather make better use of the area, for example by allowing vertical movements. The accessories need to be made of safe materials, which do not introduce extra chemicals or components into the housing environment. Inert materials are recommendable if possible or materials which are already present in the environment, such as objects made of the same wood as the bedding (e.g., aspen).

### Sensory, motor, and psychological enrichment

Sensory enrichment may include the presence of other animals, who give odor, visual or audible signals, when group housing is not possible. Objects, bedding, and nesting material give touch feelings and provision of treats are a form of taste enrichment. Shelters offer the rats the possibility to regulate the amount of light and the ambient temperature. Objects making noise give audible cues/signals and may also mask sudden and harsh noises in the laboratory animal environment, decreasing fear behavior. In addition, the sound of a radio can be used for enrichment and to mask external noises. Physical activity is quite often used in larger laboratory animals, but is possible also in small rodents (e.g., running wheels).

Psychological enrichment offers the possibility to observe, hide, explore, learn, and solve problems. The provision of shelters and shelves allows withdrawal from conspecifics and new objects offer unpredictability and the possibility to satisfy curiosity. Handling of the animals is always important for habituation but also for psychological stimulus and animal-human interactions.

### Standardization of environmental enrichment

Since the ways to enrich the environment of laboratory animals and also individual needs are dependent on the physiological state of the animal (e.g., young/old, breeding/maintenance, strain differences, social status in a group), it is virtually impossible to achieve standardization and harmonization of environmental enrichment strategies within and between laboratories. The inconsistencies with the levels of enrichment may vary even within the same facility (Hawkins, 2014). However, at least concepts of complexity, predictability and control should be kept in mind when applying an environmental strategy (Baumans et al., 2006). The complexity and variability need to be properly discussed since these will be a challenge if one wishes to undertake multi-laboratory (“phase III”) preclinical trials (Dirnagl et al., 2013).

### Large animals

Large animal stroke models have become increasingly utilized (Boltze et al., 2008), but the effect of enriched housing or neurorehabilitation for the respective species has been completely neglected. However, environmental enrichment in large animals should follow the same principles as in rodents.

## Super-enriched environment—more than environmental enrichment

The definition of enriched environment was adopted by Donald Hepp. He found that those rats that were freely allowed to explore his house had better problem-solving capabilities than laboratory-housed rats (Hepp, 1947). In animal facilities, a higher level environmental stimulation can be mimicked by providing complex, multisensory stimulation for the experimental animals (Figure 1B). Thus, a typical example of a super-enriched environment is a large, multilevel cage with more space relative to standard housing conditions. The cages can contain shelters, tunnels, ladders, and access to a running wheel to allow the animal to undertake voluntary activity and exercise. Different kinds of toys varying in shape, size, and texture are used and replaced regularly to expose the animals to novelty. An important component is social grouping, which means that animals are housed in groups of 8–12 allowing species typical behaviors such as fighting, play or sleeping together (Van Loo et al., 2004). The introduction of music, odors and different food items has also been reported as an additional stimulus (Baumans, 2005). One common practice is to put feed pellets on the bottom of the cage with bedding or different kinds of treats can be hidden into bedding or into toys, boxes, or ice cubes thus encouraging animals to seek and work for food. Although the quantity of environmental enrichment may be important for therapeutic effects (Mazarakis et al., 2014), one has to remember that more is not always better and an animal's needs and preferences should be paramount when introducing these kinds of super-enriched environments.

The design and composition of the super-enriched environment vary between laboratories and are not standardized. However, it has been reported that environmental enrichment does not increase individual variability or impair reproducibility of behavioral data (Wolfer et al., 2004). Recently, Sztainberg and Chen (2010) described a simple enrichment strategy capable of inducing a robust anxiolytic effect in mice. Detailed instructions were provided not only on how to build the environment, but also for cleaning and sterilization procedures. The voluntary exploratory activity should be observed and recorded since this can help in standardization and in quantifying the effect of housing conditions (Xie et al., 2013a). These should be feasible in stroke research as well.

With respect to animal well-being, it has been claimed that an enriched environment can allow species-specific behavior, providing animals with more control over their environment and thus reduce stress. Indeed, our observations suggest that rats housed in an enriched environment are much easier to handle, which minimizes the potentially confounding effects of emotionality and stress in sensitive behavioral testing (Benefiel and Greenough, 1998; Jones et al., 2003; Schallert et al., 2003). The animals are also more active, which is a benefit when performing tests involving spontaneous activity (e.g., cylinder tests). Thus, the environmental enrichment not only enhances the well-being of animals but also can improve the research data (Bayne and Würbel, 2014).

## Robust effect of super-enriched environment on behavioral outcome after stroke

Barbro Johansson's group introduced the concept of the super-enriched environment into the field of experimental stroke research (Johansson, 1996; Johansson and Ohlsson, 1996). Subsequently, experimental evidence has confirmed that an enriched environment can exert a robust impact on the behavioral outcome in stroke animals (Johansson, 2004; Will et al., 2004; Janssen et al., 2010). Housing in an enriched environment is also used to model stroke rehabilitation.

The efficacy of an enriched environment has been summarized in a recent meta-analysis of 21 experimental studies (Janssen et al., 2010). The results strongly support the concept that exposure to an enriched environment after a rat has suffered a cerebral ischemia could enhance the animal's sensorimotor and cognitive functions. The infarct size seemed to be slightly larger in animals recovering in an enriched environment, however this did not affect mortality. Interestingly, also exposure during the pre-ischemic time to the enriched environment has improved the recovery of motor function, spatial learning and memory without there being any reduction in brain edema or infarct volume (Xie et al., 2013b; Yu et al., 2013). More importantly, there is a report that an enriched environment can improve the rate and extent of recovery in aged stroke animals (Buchhold et al., 2007).

Although enriched environment is a powerful tool with which to enhance functional outcome after cerebral ischemia, the behavioral consequences are not completely clear. Exposure to the various novel stimuli may increase the problem solving capacity (Hepp, 1947) or engage the animals in a broader range of premorbid behaviors (Zeiler and Krakauer, 2013). A challenging environment may also prevent the development of learned non-use of the impaired limb and reliance on the non-impaired limb, a phenomenon often encountered in both experimental stroke animals and human patients (Mark and Taub, 2004; Hsu and Jones, 2006). Indeed, when skill learning of the non-paretic limb was coupled with increased dexterous use of both forelimbs in the home cage (e.g., via provision of pasta pieces, sunflower seeds, square chewing blocks), the paretic limb exhibited an improvement similar to that after focused rehabilitation (Kerr et al., 2013).

The extent to which compensation contributes to behavioral improvement promoted by enriched environment remains unclear. Knieling et al. (2009) compared the effect of an enriched environment on reaching success (quantitative) and movement patterns (qualitative) in rats subjected to a stroke. Somewhat surprisingly, the provision of an enriched environment did not promote restitution of function but did facilitate effective compensation in skilled reaching. In particular, rotating movements of the forelimb during reaching were permanently impaired and this improvement was based on a functional compensation through intensified use of the upper body.

The time when exposure to enriched environment should be started or its duration seems to be critical for recovery and achieving permanent treatment effects. While very early exposure to enriched environment may exaggerate excitotoxicity and thus infarct size (Risedal et al., 1999), there is believed to be a sensitive period when the brain is most responsive to rehabilitative training (Zeiler and Krakauer, 2013; Allred et al., 2014). Biernaskie et al. (2004) showed that housing in an enriched environment combined with task-specific training improved skilled forelimb reaching ability when the procedure was initiated between 5 and 14 days after focal ischemia in rats, but not later. Interestingly, the improvement was associated with enhanced dendritic growth in the undamaged motor cortex. The exposure duration required to achieve such permanent changes has been reported to be at least 3 weeks (Birch et al., 2013; Leger et al., 2014).

Given the complexity of enriched paradigms, it is difficult to pinpoint any single contributing factor with respect to the beneficial behavioral effects. Most likely, it is the interaction of physical exercise, sensorimotor stimulation and social components acting through common downstream mechanisms which are responsible for the improvements in the behavioral performance of animals housed in an enriched environment (Johansson and Ohlsson, 1996; Risedal et al., 2002). The importance of social interactions on stroke outcome was emphasized in a recent study conducted in mice (Venna et al., 2014). A stroke mouse paired with a healthy partner showed enhanced behavioral recovery compared with either isolated mice or a mouse paired with another stroke mouse; these beneficial effects were mediated through brain-derived neurotrophic factor (BDNF) signaling and neurogenesis. Thus, housing of sham-operated and stroke animals together in an enriched environment seems to exert an additive value.

## Effect of enriched environment on treatment outcome after stroke

The majority of stroke patients receive rehabilitation in one form or another, thus it is justified to study the combination of restorative therapies in experimental settings. Table 1 summarizes the studies in which restorative therapies have been tested in stroke rats housed in an enriched environment. In addition to anecdotal observations with different compounds (Puurunen et al., 2001b; Plane et al., 2008; Zai et al., 2011), two main approaches can be identified: pharmacotherapies acting on the noradrenergic system and cell transplantation.

**Table 1:** **Effect of therapies paired with housing in enriched environment on sensorimotor behavior in rats subjected to cerebral ischemia**.

**Treatment**	**Stroke model**	**Behavioral test**	**Effect of combined therapy**	**References**
Transplantation of fetal neocortex	distal pMCAO in hypertensive rats	rotating pole	better postural and locomotor tail position by combined therapy	Mattsson et al., 1997
Atipamezole 1 mg/kg for 10 d	tMCAO, 120 min occlusion	limb-placing, beam-walking, foot-slip	combined therapy improved limb-placing and foot-slip test immediately after administration	Puurunen et al., 2001a
Selegiline 0.5 mg/kg for 30 d	tMCAO, 120 min occlusion	limb-placing, foot-slip, Montoya's staircase	additive improvement by combined therapy in Montoya's staircase	Puurunen et al., 2001b
Transplantation of mice SVZ cells	endothelin-1	cylinder test	behavioral recovery facilitated only when cell transplants were combined with EE	Hicks et al., 2007
Retinoic acid enriched died on post-operative days 7–41	tMCAO, 90 min occlusion	cylinder, tapered/ledged beam, forelimb placing	combined treatment enhanced neurogenesis but not behavioral recovery	Plane et al., 2008
Transplantation of hESCs	distal pMCAO	cylinder test, Montoya's staircase	minor improvement in the cylinder test	Hicks et al., 2009
Amphetamine 2 mg/kg during the first post-operative week	distal pMCAO	ladder walk, skilled forelimb reaching	almost completely recovery in 8 weeks by EE, amphetamine and focused therapy	Papadopoulos et al., 2009
Atipamezole 1 mg/kg for post-operative days 2–8	distal pMCAO	ladder rung walk, Montoya's staircase	even a short term treatment when combined with EE improved recovery	Beltran et al., 2010
Inosine i.c.v. for 4 weeks starting 3 days after stroke	lesion of the caudal forelimb motor area by Rose Bengal	single pellet reaching	inosine combined with enriched environment restored forelimb use	Zai et al., 2011
Epidermal growth factor and erythropoietin i.c.v. for 2 weeks	cortical endothelin-1	cylinder test, Montoya's staircase	acceleration in recovery in the staircase	Jeffers et al., 2014

*EE, enriched environment; i.c.v., intracerebroventricular; hESCs, human embryonic stem cell-derived neural progenitor cells; pMCAO, permanent middle cerebral artery occlusion; SVZ, subventricular zone; tMCAO, transient middle cerebral artery occlusion*.

The concept of noradrenergic stimulation originates from a series of studies conducted with amphetamine, which revealed that amphetamine was effective only when paired with task-relevant experience in rats with cortical lesions (Feeney et al., 1982). This also seems to be true in stroke rats treated with amphetamine and provided with focused rehabilitative training and an enriched environment (Papadopoulos et al., 2009). More interestingly, treatment with an α_2_-adrenoceptor antagonist, atipamezole, has improved sensorimotor recovery by increasing release of noradrenaline (Puurunen et al., 2001a; Beltran et al., 2010). As in the case with amphetamine, the behavioral effects were immediate, indicating that the underlying mechanism may be reversal of remote hypometabolism (diaschisis) rather than being mediated via structural plasticity (Barbelivien et al., 2002). In addition, short-term atipamezole treatment seems to achieve a persistent and long-lasting motor improvement, but again only when paired with an enriched environment/focused rehabilitation (Beltran et al., 2010).

An enriched environment has also been combined with cell transplantation to increase cell survival and functional integration of cells, and this is then anticipated to maximize the effect of treatment. In line with this idea, neural grafting to experimental neocortical infarcts has improved behavioral outcome and reduced thalamic atrophy in rats housed in an enriched but not in a standard environment (Mattsson et al., 1997). Furthermore, the survival of a variety of cell preparations transplanted into the intact cortex was better in rats housed in enriched environment and this was to some extent reflected in a behavioral improvement seen during the first weeks after the cerebral ischemia (Hicks et al., 2007, 2009). Unfortunately, the cells were rejected in long-term studies after intracerebral transplantation even with continuous immunosuppressant drug administration.

It is not clear whether the restorative strategies described above have any direct impact on brain plasticity independently from enriched environment or possibly in synergy with enriched environment. Different repair mechanisms such neurogenesis in the subventricular zone (Hicks et al., 2007), perilesional angiogenesis (Yu et al., 2014), dendritic morphology (Johansson and Belichenko, 2002) and axonal sprouting across the midline into the denervated spinal gray matter (Papadopoulos et al., 2009; Zai et al., 2011) are activated by cerebral ischemia and these same mechanisms are further enhanced by an enriched environment. One could speculate that this interaction makes the postischemic milieu less hostile and more receptive to the provision of additional interventions. In addition, remote regions not directly affected by the ischemia may be recruited to aid in the recovery process. It is clear that more investigations are warranted to elucidate these interactions.

Why is an enriched environment not utilized more frequently, if so many restorative therapies are maximally effective when introduced in parallel with behavioral reinforcement? The obvious practical reasons are space limitations, additional personnel needed to take care of proper environmental enrichment, financial restrictions and challenges in standardization between laboratories and additional control groups may be needed perhaps complicating, even prohibiting, an adequate study design. Furthermore, one may ask the question what is a proper control group for an enriched environment—housing in single cages may mimic a deprived environment, but does it have any relevance to human life?

## Translation of experimental data

The current experimental evidence strongly suggests that an enriched environment confers an additive benefit on behavioral recovery after brain insults, most likely because of its broad impact on brain plasticity. It is tempting to speculate that the perilesional milieu may be more receptive to concomitant pharmacotherapy, cell therapy and intensive rehabilitation. The extent to which the promising data on environment enrichment can be translated into clinical practice needs to be clarified. A recent study highlighted that stroke patients living in a mixed rehabilitation unit who were exposed to an enriched environment were more likely to be engaged in activity than those not exposed to the enriched environment (Janssen et al., 2014). However, staff workload, departmental routines and personal attitudes can influence the implementation of an enriched environment (White et al., 2014). Although the importance of combined rehabilitation in cell therapy trials has been recognized in the STEPS III recommendations (Savitz et al., 2014), additional confounding variables may emerge, which complicate study design. Nonetheless, recognition of these variables and back-translation into an experimental setting should aid in assessing their possible contribution to the functional outcome in stroke patients.

### Conflict of interest statement

The authors declare that the research was conducted in the absence of any commercial or financial relationships that could be construed as a potential conflict of interest.
